# Medical cost of breast cancer services in Serbia between 2010 and 2019: national data report

**DOI:** 10.3389/fpubh.2024.1378886

**Published:** 2024-03-28

**Authors:** Nemanja Rancic, Milos Todorovic, Milos Stepovic, Stefan Vekic, Dejan Kostic, Milena Ratkovic, Svetlana Radevic, Radoje Simic, Viktorija Dragojevic Simic

**Affiliations:** ^1^Centre for Clinical Pharmacology, Military Medical Academy, Belgrade, Serbia; ^2^Medical Faculty of the Military Medical Academy, University of Defence, Belgrade, Serbia; ^3^Department of Anatomy, Faculty of Medical Science, University of Kragujevac, Kragujevac, Serbia; ^4^Faculty of Economics, University of Belgrade, Belgrade, Serbia; ^5^Department for Quality Management, Plan and Analysis, Military Medical Academy, Belgrade, Serbia; ^6^Department of Social Medicine, Faculty of Medical Sciences, University of Kragujevac, Kragujevac, Serbia; ^7^Department for Plastic Surgery, Institute for Mother and Child Health Care of Serbia Dr. Vukan Cupic, Belgrade, Serbia; ^8^Faculty of Medicine, University of Belgrade, Belgrade, Serbia

**Keywords:** breast cancer, medical costs, chemotherapy, diagnostics, pharmacoeconomic

## Introduction

Breast cancer represents a global health issue and a leading cause of high mortality and morbidity rates among women. According to the latest data from the International Agency for Research on Cancer, breast cancer was the most diagnosed malignancy among women, with 2,261,419 newly diagnosed women and around 685,000 women dying from this disease in 2020 (1). It is estimated that by 2040, there will be 3.2 million new cases of breast cancer diagnosed. Central-Eastern Europe had 158,708 new cases of breast cancer (7.0% of world region) and 51,488 deaths (7.5% of world region) in 2020, with dominant age group of ≥50 years (2). Breast cancer also causes significant morbidity and mortality in Serbia. Based on the same database, there were 6,724 newly discovered cases (13.7% of all tumors), while the number of deaths was 2,342 (8.3% of all tumor-related deaths) in 2020 (3). Moreover, Serbia is the country with the continual annual increase of the standardized incidence rate, as a leading cause of cancer mortality in women (4). In 1990 to 2019 with forecasts up to 2030, revealed that among 11 countries in this region, Serbia has the highest disability-adjusted life years (DALYs) rate (671.00 per 100,000) and the highest years of life lost (YLL) rates (623.00 per 100,000) (5). Additionally, the highest DALYs rate (731.89 per 100,000) and highest YLL rate (674.34 per 100,000) in 2030 is forecasted for Serbia, and together with the fact that third highest incidence rate of breast cancer in the Balkan region in 2019 is documented in Serbia suggest ineffective prevention programs and premature mortality (5).

Multidisciplinary treatment for breast cancer, involving a combination of surgical removal, radiation therapy, and anticancer drugs, can be highly effective, particularly when the tumor is detected in early clinical stages (6, 7). The survival rate for patients diagnosed in early stages (I and II) is 85–95%, while for those diagnosed in late stages (III and IV), it ranges from 30–70% (8). Risk factors for breast cancer can be divided into modifiable and non-modifiable factors (9). Non-modifiable factors include age, positive family history, genetic mutations BRCA1 and BRCA2, early menarche, and late menopause. Modifiable factors include improper nutrition and obesity, smoking, alcohol consumption, lack of physical activity, and oral contraceptives (9).

Despite significant progress in the treatment and survival rates, the incidence of breast cancer continues to rise in both developed and developing countries, imposing a substantial economic burden on healthcare systems (10, 11). The economic burden of breast cancer treatment has been estimated in various countries. In the United States, the total cost of breast cancer treatment in 2020 was $20.5 billion. In other countries like South Korea and Spain, expenditures for treatment amounted to $940.75 million and €518 million, respectively (8).

On-time diagnosis of breast cancer can save many years of woman life and the preventive program for the early detection and treatment is available in every country. However, there are difficulties in reaching proper medical health care with underlining economic allocation problems for health sectors in differently developed countries. Many of the medicines used to treat early breast cancer are effective and available at low cost (12). However, systematic reviews that analyze the cost of breast cancer diagnosis or treatment in low- and middle-income countries are limited, as they consider the cost-effectiveness of specific chemo- and biologic therapies or focus on specific geographic regions (13).

Although breast cancer possesses a considerable economic burden on healthcare systems in many countries, its economic impact has not been studied in Serbia enough. The aim of this data report is to describe the overall medical costs for breast cancer patients over a 10-year period (from 2010 to 2019) and to compare the costs of three major groups of anticancer drugs.

## Methodology

This is a retrospective, observational cost study that describes the different direct medical expenditures accrued by the breast cancer patients in a 10-year period (2010–2019). Records of breast cancer patients who received medical care at any Serbian hospital were included only, with no regards of the cancer stage at the time of treatment or diagnosis. The 2019 year was taken as the last observed year because it was the year when the spending patterns were changed due to COVID pandemic.

National Health Insurance Fund (NHIF) was the main source of data for this study (https://www.eng.rfzo.rs/index.php). The researchers were provided with data consisting of information on demographics, medical services provided and the associated expenses with exclusion of personal health identifiers. In Serbia, anticancer drugs and radiotherapy can be administered in the public hospitals only, fully covered by the NHIF, and this ensures the validity of our data.

The medical services were presented and divided into the procedures and expenditures associated with diagnosis, therapy, inpatient/outpatient care, physician examination, preparation and administration of drugs, nursing care, and other related medical services.

The use and costs of all breast cancer medication were derived from the publication “Marketing and Consumption of Medical Products for Human Use,” published annually by the Medicines and Medical Devices Agency of Serbia (accessed at https://www.alims.gov.rs/latin/o-agenciji/publikacije/).

The out-of-pocket (OOP) expenses (OTC preparation, dietary supplements, vitamins, minerals, etc.), loss of productivity related costs and costs associated with premature death were not available and were not within the scope of this report.

The principles of ICH Good Clinical Practice were strictly followed in this study.

### Cost of breast cancer in Serbia

The government of Serbia commitment in fighting malignant diseases is evident through collaboration with the World Bank. The Ministry of Health (MOH**)** invested almost 70% of additional financing from the World Bank's Second Health Project ($ 21 million) in radiology infrastructure for improving cancer diagnosis and treatment in 2018. Oncology is the second largest disease expenditure ($ 222 million), just following cardiovascular diseases which consumed $301 million in 2017 (14).

According to NHIF, the average public expenditure for an oncology patient is $ 860 per year, 58% of which covers health services, and 42% covers drugs and medical supplies. Due to the inefficiencies in the provision of care, such as long time on waiting lists, patients who can afford it often turn to the private health sector, mainly for diagnosis and follow-up screening. Key informant estimates suggest that this oncology related OOP spending averages approximately $20 per month per patient (15, 16).

The market for oncology drugs represents only 10% of the whole pharmaceutical market in Serbia, by far the lowest share in the region, in comparison to Bulgaria, Slovenia, Italy and Croatia. Serbia also had the lowest per capita expenditure on oncology drugs ($13) among its neighbors and was far behind European countries in adoption of innovative therapies, such as bio-, immuno-, and hormone-therapies (14). The analysis of adoption of the latest 43 innovative drugs in Europe for oncology ranks Serbia as the last one with only one innovative therapy on the reimbursement list while the average in EU is 24 (17).

Late diagnosis is one of the key challenges of the Serbian oncology program. In 52.5% of cases, the malignant disease in Serbia is diagnosed in an advanced stage (III-b or IV) with regional and distant metastases (18). Since the costs of breast cancer treatment is progressively increasing with new expensive therapy options and better progression free survivals and overall survivals, there is an exponential increase in treatment costs, especially for later stages of illness (19).

Serbia had one the lowest number of computed tomography (CT) scanners per 100,000 inhabitants in 2016 along with Hungary (0.9) and the lowest number of magnetic resonance imaging (MRIs) per 100,000 people (0.3), after Hungary (0.4) (20). Serbia moves toward the EU standard concerning radiotherapy. Between 2016 and 2019, the MOH invested in 15 linear accelerators for radiotherapy treatment, significantly reducing time on waiting lists, therefore increment in patient survival rate are expected to take place by 2022 (14).

A low- and middle-income country have higher rates of breast cancer mortality in comparison to high-income countries and with it comes the higher costs which affects the cancer care delivery, policy and strategies of countries which are crucial components for accessing the good health care (13). Focusing on the EU-27, breast cancer is the most diagnosed cancer with over 360,000 new female cases (13.3% of all cancer diagnoses), followed by colorectal and prostate, and third overall when considering the most common cause of death (21). The total costs of healthcare provided for breast cancer patients in Serbia was found to be between 34.53 million € in 2010 and 25.58 million € in 2019, and these concern only direct costs (Table 1). Data concerning Vojvodina, northern region in Serbia, for 2019 indicated that the total costs for breast cancer were 15 million €, but direct costs accounted for 5 million €, i.e., 34% (23). It is estimated that in 2019 Serbia had 6,963,764 inhabitants, while Vojvodina had 1,852,093 inhabitants. Therefore, breast cancer was also great financial burden for health system in Vojvodina, like in Serbia, as a whole.

**Table 1:** Healthcare costs of breast cancer in Serbia (€, yearly, 2010-2019).

	**2010**	**2011**	**2012**	**2013**	**2014**	**2015**	**2016**	**2017**	**2018**	**2019**
Total healthcare cost	34,539,437	33,001,797	29,283,931	29,133,160	25,687,397	24,759,078	27,079,628	27,310,321	24,632,120	25,584,548
Diagnosis cost	2,839,918	3,075,768	2,668,558	2,828,918	3,301,915	3,648,928	4,084,043	4,274,096	4,320,979	4,501,519
% Total	8.2	9.3	9.1	9.7	12.9	14.7	15.1	15.7	17.5	17.6
Laboratory	1,523,792	1,697,532	1,581,170	1,819,405	973,390	1,136,915	1,239,555	1,370,165	1,464,203	1,561,230
Tumor Markers- Breast cancer	187,433	244,491	209,530	243,999	91,210	124,565	136,202	149,059	153,561	155,316
% Total	12.3	14.4	13.3	13.4	9.4	11.0	11.0	10.9	10.5	9.9
Radiology (classic, MRI, CT, USI)	874,823	914,528	697,044	600,552	942,673	1,075,244	1,204,681	1,278,859	1,329,839	1,348,468
Nuclear medicine	204,743	220,613	179,985	190,235	352,692	350,289	554,472	538,335	500,760	510,424
Endoscopy (diagnosis and therapy)	5,461	4,535	3,527	3,120	4,829	4,321	6,899	8,374	6,753	6,604
Biopsy and surgery (incision)	101,980	106,221	92,338	100,820	493,306	478,625	527,046	548,939	528,075	562,117
Other	129,118	132,338	114,493	114,785	535,023	603,534	551,391	529,423	491,349	512,677
Therapy cost	25,248,307	23,643,024	21,314,520	20,979,048	16,770,879	15,163,112	17,016,071	16,949,372	14,422,483	15,474,728
% Total	73.1	71.6	72.8	72.0	65.3	61.2	62.8	62.1	58.6	60.5
Medication	20,523,187	18,749,213	17,159,209	17,193,473	12,958,559	11,786,411	13,274,663	13,405,777	11,518,109	12,859,210
Medical devices	707,545	822,932	712,787	682,161	455,222	440,541	503,817	544,573	540,641	538,264
Surgery	645,696	667,262	546,252	554,554	821,535	850,442	825,224	829,832	869,105	916,825
Radiotherapy	2,976,321	2,873,768	2,620,373	2,414,406	2,469,865	2,003,247	2,339,985	2,090,452	1,409,104	1,071,984
Interventional radiology	15,089	18,101	8,422	16,846	8,653	6,513	6,145	8,582	8,345	11,060
Other	380,470	511,748	267,477	117,608	57,046	75,958	66,237	70,155	77,180	77,385
Inpatient days	2,004,722	1,970,324	1,745,426	1,724,321	1,193,760	1,112,379	1,098,432	1,085,666	1,076,667	949,326
% Total	5.8	6.0	6.0	5.9	4.6	4.5	4.1	4.0	4.4	3.7
Outpatient days	370,684	428,914	416,768	434,560	273,905	291,311	319,918	347,099	294,256	122,667
% Total	1.1	1.3	1.4	1.5	1.1	1.2	1.2	1.3	1.2	0.5
Physician examination	1,444,265	1,469,474	1,250,214	1,242,090	1,437,764	1,580,789	1,484,598	1,523,364	1,558,289	1,514,021
% Total	4.2	4.5	4.3	4.3	5.6	6.5	5.5	5.6	6.3	5.9
Administration	4,529	3,470	1,242	534	42	23	20	20	27	15
% Total	0.0131	0.0105	0.0042	0.0018	0.0002	0.0001	0.0001	0.0001	0.0001	0.0001
Nursing care	562,372	493,371	385,659	346,099	928,140	1,128,836	1,167,763	1,157,271	978,521	810,729
% Total	1.6	1.5	1.3	1.2	3.6	4.6	4.3	4.2	4.0	3.2
Other	2,064,639	1,917,454	1,501,543	1,577,589	1,780,992	1,833,698	1,908,782	1,973,432	1,980,898	2,211,544
% Total	6.0	5.8	5.1	5.4	6.9	7.4	7.0	7.2	8.0	8.6
National inflation rate (%)^*^	6.14	11.14%	7.33%	7.69%	2.08%	1.39%	1.12%	3.13%	1.96%	1.85%

All healthcare costs are expressed in euros. All data coming from the National Health Insurance Fund. ^*^Data from Serbia Inflation Rate 1995-2024 (22).

In highly developed countries like US, projections of the cost of cancer care pointed to rose from $26.8 billion in 2015 to $29.8 billion in 2020, as a 11.2% increase (24, 25). Although the United States (US) is considered a very developed country, it is also considered one of the countries whose healthcare system is one of the least effective—the COVID-19 pandemic has made this very evident. Countries like Serbia where the Unified Health System (SUS) exists had more effective results, even with a lower per capita value during the pandemic compared to the United States (US). This is justified because in countries like Serbia, the Health System is universal and comprehensive (26), therefore in crisis situations they tend to respond better and be more resilient and responsive.

Breast cancer has the highest treatment cost of any cancer, accounting for 14% of all cancer treatment costs (27). The costs for medical services and prescription drugs in breast cancer patients was $26.2 billion and $3.5 billion, respectively in 2020 in US. The average per-patient costs for medical services were highest for the end-of-life phase (year before death from cancer, $76,100), followed by the initial care phase (first year after diagnosis, $35,000), and continuing care phase (the time in between these two phases, $3,500) (27).

Cancer costs the EU €126 billion in 2009. Lung cancer had the highest economic costs (€18.8 billion, 15% of overall cancer costs), followed by breast cancer (€15.0 billion, 12%) (28). Considering healthcare costs only, breast cancer came in the first place. Newly diagnosed cancer patients from the Central Serbian region, regarded as representative of national cancer incidence and prevalence rates, were included in retrospective data-based analysis regarding two-year period (2010–2011) (29). This investigation of end-of-life costs of medical care for advanced stage cancer patients indicated that the average costs per patient were highest (13,114.10 €) and lowest (4.00 €) in breast cancer and melanoma.

We further analyzed the expenditures associated with the diagnosis, therapy, inpatient/outpatient care, physician examination, drug preparation/administration, nursing care, and other breast cancer patient services. The NHIF database breaks down diagnostic services into the laboratory, radiology services (classic ones, magnetic resonance imaging, computed tomography scan, ultrasound imaging, interventional services), nuclear medicine services, endoscopic services (examinations and treatments), biopsy (including surgery), and other.

Diagnostics of breast cancer in our research increased from 2.83 million € in 2010 to 4.5 million € in 2019. We found different trends regarding the costs of these services—radiology, nuclear medicine, biopsy, endoscopy and other ones, with relatively steady cost of laboratory (Table 1).

Within the observed 10-year period, the national inflation rate has significantly fluctuated from 2010 to 2013, and in 2011 it amounted 11.14% (Table 1). After that, the national inflation rate has slight fluctuations from 2014 to 2019, in the range from 1.12% to 3.13%. Inflation continues to be a major headline in many countries with each industry branches and sector feeling the repercussions of the nation's economic climate. However, although the inflation rate was the highest in Serbia in 2011, the total costs were not significantly higher compared to other years (Table 1). In the following years, the total healthcare costs of breast cancer in Serbia had more or less decreasing trend. If it is known that, as a rule, inflation or specific price indices are included in the next year's price determination, one could expect the opposite scenario. However, the healthcare industry has traditionally been described as recession-proof, and indeed, from a healthcare perspective, the cost of medical services has lagged slightly compared to other parts of the economy (30). Therefore, it can be considered that inflation in the 10-year follow-up period did not have a significant impact and was not reflected in the growth of the total treatment costs of these patients in our country. This can be explained by the fact that in Serbia, health care is mostly financed from the state budget, so with the intervention of state, health services have a relatively stable price (26).

The costs of breast cancer diagnosis accounted for 8.2% of the total breast cancer costs in 2010 and showed an increment up to 17.6% in 2019 (Table 1). The laboratory services as a part of the diagnostic costs were the most expensive (1.43 million € on average), while the tumor breast markers showed steady percent of costs with roughly 12% of all cost from laboratory analysis during follow-up period. Radiology, nuclear medicine, endoscopy, biopsy, and other services increased from 2010 to 2019 by 1.54 times, 2.5 times, 1.2 times, 5.5 times, and 3.9 times, respectively.

Trend in therapy costs for breast cancer was in contrast with diagnostic costs. A decreasing trend within 10-year period, from 25,24 million € in 2010 to 15,47 million € in 2019 was observed (12.6%), although considering absolute costs in Euros, a larger sum invested in breast cancer patients was used for therapy comparing with the funds invested in diagnostics. Therefore, it appeared that in this period, the focus of the health authorities was on the prevention and early diagnosis of the breast cancer, since it was obviously that there were ineffective preventive programs and premature mortality in our country (5).

Therapy costs were divided into the subgroups in the following order—medication, medical devices, surgery, radiotherapy, interventional radiology, and others (Table 1). All kinds of costs for the breast cancer therapy showed a decreasing trend over the observed period, except for surgery costs which increased by about 42%. Overall, the most expensive was medication, which included all kind of drugs used for breast cancer treatment, but also with continuing decrease over the follow-up period.

We found that the relative costs of inpatient days for breast cancer were on the continuous decline, from 5.8% in 2010 to 3.7% in 2019, as like the costs of outpatient days which roughly decreased double (from 1.1% in 2010 to 0.5% in 2019) (Table 1). The physician examination costs began to rise, from 4.2% in 2010 to 5.9% in 2019, while the relative cost of nursing care was from 1.6% (2010) to 3.2% (2019). Collectively, the costs of inpatient days, outpatients' days, physician examination, and nursing care increased from, 12.67% to 13.26% of the total breast cancer costs from 2010 to 2019.

### The cost structure of breast cancer therapy

Total breast cancer therapy costs varied from 25.2 million € in 2010 to 15.5 million € in 2019, of which total chemotherapy accounted for 74.7% to 79.68% of the total therapy costs, respectively (Tables 1, 2).

**Table 2:** Anticancer drugs costs for breast cancer in Serbia (€, yearly, 2010–2019).

	**2010**	**2011**	**2012**	**2013**	**2014**	**2015**	**2016**	**2017**	**2018**	**2019**
Anticancer drugs	18,862,448	17,583,089	16,311,769	16,355,649	12,357,407	11,247,633	12,658,712	12,603,289	10,817,648	12,331,470
Biological therapy	10,276,136	10,693,866	11,670,726	12,257,596	9,036,255	9,385,839	10,527,925	10,153,320	8,595,525	10,574,905
% Total	54.5	60.8	71.5	74.9	73.1	83.4	83.2	80.6	79.5	85.8
Hormonal therapy	4,725,464	3,687,075	2,686,628	1,971,798	1,133,303	581,780	659,925	904,523	650,400	328,785
% Total	25.1	21.0	16.5	12.1	9.2	5.2	5.2	7.2	6.0	2.7
Cytotoxics and antimetabolites	3,860,849	3,202,148	1,954,415	2,126,255	2,187,849	1,280,013	1,470,862	1,545,447	1,571,723	1,427,780
% Total	20.5	18.2	11,9	13.0	17.7	11.4	11.6	12.3	14.5	11.6

All healthcare costs are expressed in euros. All data coming from the National Health Insurance Fund.

Costs of anticancer drugs (total chemotherapy) were divided into the subgroups in the following order—biological therapy, hormonal therapy and cytotoxics and antimetabolites (Table 2). During follow-up period, great changes have occurred concerning biological therapy which accounts for 85.8 % of the total chemotherapy costs in 2019, i.e., it increased for 31.3%. However, that large relative increase in costs is largely the result of a decrease in overall costs for anticancer drugs from 2010 to 2019. On the contrary, large decrease of sum invested in hormonal therapy has been noticed, from 25.1% in 2010 to 2.7% in 2019. Costs of cytotoxics and antimetabolites decreased, and it accounted for only 11.6% of all anticancer drug costs in 2019 (Table 2).

Serbia has two health technology assessment (HTA) committees, both with limited capacity to assess population needs and to conduct “tailored” cost effectiveness analyses. One of them is within the MOH and another is housed by the NHIF, that is Central Drug Committee (CDC) (31). The CDC primarily assesses the HTA of pharmaceutical companies, submitted as a part of their application for inclusion of new drugs on the NHIF reimbursement list. After conducting budget impact analysis, the CDC submits recommendations for final approval to the MOH. The ultimate criteria for accepting a drug for national reimbursement list is the budget availability. Serbian GDP per capita and other economic indicators are too low to make innovative therapies appearing cost-effective using the standard National Institute for Health and Care Excellence (NICE) style for HTA assessment (32). Despite the existence of two HTA committees, the introduction of new drugs and medical devices is carried out by *ad hoc* evaluations that largely take into account the budgetary impact, without sufficient assessment of the optimal cost/benefit ratio (33).

Limitations of the study: The study did not cover the period from 2020 onwards, due to COVID-19 pandemic and, unfortunately, we suppose that there were some changes in making diagnoses and therapy opportunities for these patients. According to Italian researchers' estimates undetected breast cancer cases rate ranges between 5.01 and 11.53% due to the lockdown (34). Therefore, higher health care costs and worsening of long-term outcomes of breast cancer is something that all health systems, including the one in Serbia, would face in the near future. Our study is retrospective observational study. However, we believe that its importance corresponds to the facts that included data relate to the national level and for a whole decade follow-up period.

## Conclusion

The number of breast cancer cases is increasing every year, while the total funds allocated for these patients' healthcare decreased by almost 26% from 2010 to 2019. Although the costs for breast cancer therapy in absolute numbers were significantly higher than those invested in diagnostics, in the period from 2010 to 2019, a significant reduction took place. In contrast, during the same period, there was an increase in investments in the prevention and early diagnosis of breast cancer, a primary goal for the healthcare authorities. Since the funds invested in the financing of biological therapy did not change significantly during the observed period, and there was a decrease in total investments for anticancer drugs, costs for this therapy increased only marginally. The costs of nursing care and physician examinations increased by 1.6% and 0.7% respectively. The costs of hospital and outpatients' care for this category of patients decreased, although this was more prominent for outpatients' care. These results are relevant to helping health care planners and policymakers allocate resources in an optimal way. However, studies on service utilization patterns and costs of cancer care are greatly needed for the countries in transition, like Serbia, especially after the pandemic crisis that had a substantial impact on healthcare costs and risks. Further investment in prevention, such as application of MicroRNAs (miRNAs), important regulators of gene expression, and potentially powerful specific tool as a cancer biomarker, is greatly needed.

## Data availability statement

The raw data supporting the conclusions of this article will be made available by the authors, without undue reservation.

## Author contributions

NR: Conceptualization, Formal analysis, Methodology, Project administration, Resources, Software, Supervision, Validation, Writing—original draft. MT: Data curation, Investigation, Methodology, Validation, Writing—original draft. MS: Formal analysis, Investigation, Methodology, Supervision, Validation, Visualization, Writing—original draft. SV: Data curation, Project administration, Software, Visualization, Writing—original draft. DK: Formal analysis, Project administration, Software, Validation, Writing—original draft. MR: Data curation, Writing—original draft. SR: Investigation, Methodology, Validation, Writing—original draft. RS: Investigation, Methodology, Validation, Writing—original draft. VDS: Conceptualization, Data curation, Formal analysis, Methodology, Supervision, Writing—original draft.
